# 355110- Potentialities and challenges of *stricto sensu* graduate studies in health: A qualitative meta-synthesis

**DOI:** 10.17533/udea.iee.v43n1e16

**Published:** 2025-04-29

**Authors:** Carolina Cassiano, Laura Andrian Leal, Mári Andrade Bernardes, José Carlos Marques de Carvalho, Silvia Helena Henriques

**Affiliations:** 2 Nurse, Ph.D in Sciences. Doctor Professor. Email: laura.andrian.leal@usp.br https://orcid.org/0000-0003-3549-2538 Universidade de São Paulo Brazil laura.andrian.leal@usp.br; 3 Degree in Textile and Fashion. Master of Sociology. Email: maribernardesusp@gmail.com. https://orcid.org/0000-0002-8563-8980 Universidade Federal de São Carlos Brazil maribernardesusp@gmail.com; 4 Nurse, Ph.D in Nursing Science. Associate Professor. Email: zecarlos@esenf.pt https://orcid.org/0009-0007-2724-0695 Nursing School Portugal zecarlos@esenf.pt; 5 Nurse, Ph.D in Nursing. Associate Professor. Email: shcamelo@eerp.usp.br https://orcid.org/0000-0002-8391-8647 Universidade de São Paulo Brazil shcamelo@eerp.usp.br; 6 Nursing School, University of São Paulo, Ribeirão Preto, São Paulo, Brazil. https://orcid.org/0000-0003-2089-3304 Universidade de São Paulo Nursing School University of São Paulo Ribeirão Preto São Paulo Brazil; 7 Federal University of São Carlos, São Paulo, Brazil. Universidade Federal de São Carlos Federal University of São Carlos São Paulo Brazil; 8 Nursing School, Porto, Portugal. Nursing School Nursing School Porto Portugal

**Keywords:** education, education, graduate, teaching, students, research, graduate programs in health., educación, educación de posgrado, enseñanza, estudiantes, investigación, programas de posgrado en salud., educação, educação de pós-graduação, ensino, estudantes, investigação, programas de pós-graduação em saúde.

## Abstract

**Objective.:**

To analyze the potentialities and challenges of *stricto sensu* graduate programs in the health field from the perspective of post-graduate students and graduates.

**Methods.:**

This study is a qualitative meta-synthesis analyzing 23 studies selected from the following databases: BDENF, LILACS, MEDLINE via PubMed, PsycINFO and Scopus in Spanish, English and Portuguese, published between 2002 and 2022. Data were analyzed using the constant comparative analysis technique.

**Results.:**

The synthesis identified the potentialities and challenges of *stricto sensu* graduate studies across four domains: personal, academic, professional, and social. Key potentialities included the development of research skills, the production of relevant studies, the training of highly qualified professionals, and interdisciplinary collaboration. However, significant challenges were also noted, such as time management difficulties, high academic demands, competitiveness, workload overload, financial constraints, and professional undervaluation.

**Conclusion.:**

The qualitative studies reviewed highlight both the potentialities and challenges of *stricto sensu* graduate programs in the health field, emphasizing their impact on personal, academic, professional, and social aspects. It is essential for training institutions to develop and implement strategies that support graduate students in overcoming the challenges inherent in this formative process.

## Resumen

**Objetivo.:**

Analizar las potencialidades y los desafíos de los cursos de posgrado *stricto sensu* en el área de la salud, desde la perspectiva de estudiantes de posgrado y egresados.

**Métodos.:**

Metasíntesis cualitativa, a través de la cual se realizó el análisis de 23 estudios, seleccionados de las bases de datos: BDENF, LILACS, MEDLINE vía PubMed, PsycINFO y Scopus en español, inglés y portugués, publicados entre 2002 y 2022. El análisis de datos se llevó a cabo mediante la técnica comparación constante.

**Resultados.:**

La síntesis permitió destacar potencialidades y desafíos de los estudios de posgrado *stricto sensu* en los siguientes ámbitos: personal, académico, profesional y social. Entre las potencialidades, se destacaron el desarrollo de competencias de investigación, estudios relevantes, la formación de profesionales calificados y la colaboración interdisciplinaria. No obstante, existen desafíos como dificultades en la gestión del tiempo, altas exigencias académicas, competitividad, sobrecarga, dificultades financieras y desvalorización.

**Conclusión.:**

Los estudios cualitativos exploraron las potencialidades y los desafíos de los programas de posgrado *stricto sensu* en el área de la salud, con énfasis en este estudio en las dinámicas personales, académicas, profesionales y sociales del campo. Resulta relevante para los centros de formación la creación y gestión de estrategias que inciten a los estudiantes de posgrado a enfrentar, sobre todo, los desafíos presentes en este proceso formativo.

## Resumo

**Objetivo.:**

Analisar as potencialidades e os desafios dos cursos de pós-graduação *stricto sensu* na área da saúde, na perspectiva de pós-graduandos e egressos.

**Métodos.:**

Trata-se de uma metassíntese qualitativa, por meio da qual foram analisados 23 estudos, selecionados nas seguintes bases de dados: BDENF, LILACS, MEDLINE via PubMed, PsyINFO e Scopus em espanhol, inglês e português, publicados entre 2002 e 2022. Os dados dos artigos foram analisados utilizando a técnica de análise de comparação constante.

**Resultados.:**

A síntese permitiu evidenciar as potencialidades e os desafios da pós-graduação *stricto sensu* nos âmbitos: pessoal, académico, profissional e social. Dentre as potencialidades, destacaram-se o desenvolvimento de competências de pesquisa, estudos relevantes, formação de profissionais qualificados e colaboração interdisciplinar. No entanto, há desafios, como dificuldades na gestão do tempo, alta exigência acadêmica, competitividade, sobrecarga, dificuldades financeiras e desvalorização.

**Conclusão.:**

Os estudos qualitativos exploraram as potencialidades e os desafios dos programas de pós-graduação *stricto sensu* na área da saúde, destacando-se nesta pesquisa as dinâmicas pessoais, acadêmicas, profissionais e sociais do campo. É relevante que os centros formadores criem e gerenciem estratégias que auxiliem os pós-graduandos a enfrentarem, sobretudo, os desafios presentes nesse processo formativo.

## Introduction

*Stricto sensu* graduate programs, including master’s and doctoral degrees, aim to train future educators and researchers, significantly contributing to society through the expansion of higher education and the promotion of research. In Brazil, this expansion has been evident and has contributed to advancements in knowledge, science, innovation, and technology, which are critical aspects for economic and social achievements.^1^ Thus, driven by demand and supported by governmental policies, an increasing percentage of health professionals have applied to graduate programs.^2^ In this regard, it is known that graduate programs in the health field are essential for knowledge production as well as for addressing societal needs and enhancing professional skills in their respective fields.^3^ Within this context, the potentialities and challenges within graduate education in the health field raise interest for those undertaking such programs. The reasons for choosing a *stricto sensu* graduate program may be related to the pursuit of knowledge or career advancement.^4^ Research has shown that financial success, salary increases, and the opportunity to perform meaningful work for society are also significant motivational factors in this formative process.^5^^,^^6^It should be emphasized that pursuing a *stricto sensu* graduate degree holds notable importance for health professionals, as research has a relevant societal impact and influences students’ learning styles, promoting critical analysis, skill acquisition, information literacy, and evidence-based practice, which is crucial in health services.^7^ Such aspects represent potential benefits for intellectual development, competency building, and career progression. However, to acquire academic skills and advance professionally, research opportunities, academic support, and appropriate professional positions aligned with one’s qualifications must be encouraged.^8^

It is evident that entering a *stricto sensu* graduate program can lead to significant changes, generating expectations regarding this new phase of life and its academic demands.^9^ Moreover, from a global perspective, graduate education faces numerous challenges;^10^ after enrollment, students must undertake a series of tasks, such as the research process, which involves identifying a topic, collecting and analyzing data, writing, and even publishing articles.^2^ It is worth noting that academia has faced a mental health crisis, particularly affecting early-career researchers.^11^ Challenges may also be related to the research execution process, presenting barriers that need to be overcome to achieve success. Nevertheless, some students are unable to overcome these obstacles and may feel isolated during the process. Moreover, ineffective communication, unsupported responsibilities, lack of counseling and guidance, and misunderstanding of the research process can pose significant challenges during *stricto sensu* graduate studies.^12^Thus, the current transformation of the labor market has influenced the construction of professional trajectories and the development of career management strategies and personal goals. Given this scenario, health professionals constantly seek improvement to meet labor demands, despite the challenges that may arise. Furthermore, the post-industrial society, economic instability, and restructuring within health organizations require qualification and adaptation of professionals to the work context, impacting career trajectories and potentially altering the perspectives of these health workers and their entry into *stricto sensu* graduate programs. 

Analyzing the potentialities and challenges of master’s and doctoral programs, whether academic or professional, in the health field is justified by the fact that this is a pressing issue requiring qualitative exploration, given the limited data on the topic examined through this methodological approach. Moreover, it becomes relevant to understand how these potentialities contribute to personal, professional, and collective development, encompassing graduate programs and society as a whole. It is also necessary to analyze the challenges faced by these students, enabling the adoption of management strategies that overcome or minimize these issues. In light of the above, the objective of this study is to analyze the potentialities and challenges of *stricto sensu* graduate programs in the health field from the perspective of current students and alumni.

## Methods

This is a qualitative meta-synthesis, which consists of synthesizing and interpreting the results of qualitative studies within a specific area to achieve a broader understanding and formulate new findings that go beyond existing studies.^13^ Thus, the proposal by Sandelowski and Barroso was adopted,^13^ which follows a set of steps including formulating the research question, identifying and systematically selecting the articles to be analyzed, evaluating the chosen articles, extracting relevant data, and synthesizing the results. To ensure data reliability and methodological transparency in this review, the protocol was submitted to the Open Science Framework (OSF/Center for Open Science/USA) for registration purposes, with the DOI identification: 10.17605/OSF.IO/SPZQT. It is worth noting that a search conducted in May 2023 in the registry did not locate any protocols on this topic. It should also be emphasized that recommendations outlined in the Enhancing Transparency in Reporting the Synthesis of Qualitative Research (ENTREQ)^14^ were used to draft the qualitative synthesis.

A literature search was conducted to identify original studies with a qualitative approach addressing the theme, aiming to enhance understanding of the subject based on the following guiding question: “What are the qualitative evidences regarding the potentialities and challenges of *stricto sensu* graduate programs in the health field, from the perspective of current students and alumni?” For this study, five databases were utilized: Nursing Database (*Base de Dados da Enfermagem* - BDENF), Latin American and Caribbean Literature in Health Sciences (*Literatura Latino-Americana e do Caribe em Ciências da Saúde* - LILACS), MEDLINE via PubMed (United States National Library of Medicine), PsycINFO (American Psychological Association), and Scopus (Elsevier).

Original articles that employed qualitative methodologies, as well as descriptive and exploratory studies involving qualitative data analysis, were considered for inclusion. These studies were required to have been conducted with students currently enrolled in and pursuing a master’s or doctoral program, whether academic or professional, or with alumni, and the graduate program had to be in the health field. Studies were included if their participants were graduate students pursuing a master’s and/or doctoral degree in the health field. The inclusion of studies with graduate students was based on their firsthand experience of the current realities of graduate education, while studies with alumni were included because, despite having completed their programs, they also experienced this formative process and possessed knowledge of the academic context and relevant experiences. Studies published in Spanish, English, or Portuguese between 2002 and 2022 were included. Additionally, mixed-methods studies in which the qualitative analysis of results was presented separately from quantitative data were also incorporated. Studies that did not align with the Population, Context, and Concept (PCC) strategy^15^ were excluded: P - Population (master’s students, doctoral students, or alumni), C - Concept (qualitative studies addressing potentialities and/or challenges of *stricto sensu* graduate programs), and C - Context (health field), consistent with the study objectives and research question.

Data collection was performed independently by two reviewers between June and July 2023. The search strategy included keywords based on the pre-established PCC acronym.^15^ Thus, the strategy was constructed using Health Sciences Descriptors (*Descritores em Ciências da Saúde* - DeCS) and Medical Subject Headings (MeSH), along with additional keywords to recruit more studies encompassing the theme, combined using Boolean operators (AND and OR): ‘Master’s Degree/Mestrado’, ‘Mastering/Mestrando’,‘PhD/Doctorate/Doutorado’, ‘Doctorate student/PhD student/Doutorando’, ‘Postgraduate/Pós-graduação’, ‘Graduate education/Ensino de Pós-Graduação’, ‘Education, Graduate/Educação de Pós-Graduação’, ‘Health/Saúde’, ‘Health Personnel/Pessoal de Saúde’, ‘Health Post graduate Programs/Programas de Pós-Graduação em Saúde’.

The Preferred Reporting Items for Systematic Reviews and Meta-Analyses (PRISMA)^16^ was also used to describe the process of searching the scientific literature. The articles were transferred to the Rayyan™ software^17^ to carry out the selection process and identify any duplicates. The included articles were re-read by two authors, and the data extraction process occurred through a spreadsheet designed based on the research question adopted in this meta-synthesis and the general characteristics of the studies: year of publication, authors, country of origin, journal, methodological aspects (study design, participants, and data collection procedures), and the results presented on the topic.

The quality of the articles was assessed independently by two reviewers using the Critical Appraisal Skills Programme (CASP).^18^ This is an instrument consisting of a checklist with ten questions aimed at evaluating the description and relevance of the objectives of qualitative studies. Several aspects of the articles were evaluated, including the appropriateness of the qualitative methodology, study design, recruitment strategy, data collection, adequacy of the researcher-participant relationship, ethical considerations, rigor of data analysis, presentation of results, and the study’s contributions. Any discrepancies between the reviewers were addressed and resolved through in-person discussions until a consensus was reached. To perform the data analysis and develop the synthesis, the constant comparison analysis technique proposed by Sandelowski and Barroso^13^ was applied. For the development of categories and interpretive synthesis, the properties and variations indicated by the results were considered, as well as the underlying concepts and explicit or implicit conceptual relationships in the data. Additionally, concepts from the literature were incorporated to integrate the findings into a central concept. The obtained codes were organized into taxonomies according to their similarities. The coding and categorization process was carried out by two authors, who discussed discrepancies together. To ensure the validity of the synthesis, three other authors with experience in qualitative research carefully reviewed the accuracy of the codes, taxonomies, and concepts related to the categories.

## Results

**Figure 1:**
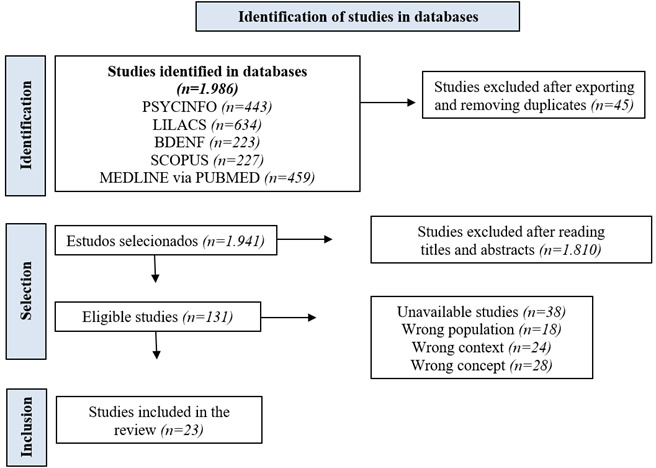
Flowchart of the article selection process for the qualitative meta-synthesis

A total of 1,986 studies were identified across the five databases used, and from this total, 45 duplicate articles were excluded, leaving 1,941 studies. Subsequently, two independent reviewers conducted a title and abstract screening, considering the inclusion and exclusion criteria. In all, 131 articles were initially selected as meeting the inclusion criteria. Furthermore, a full-text review of these studies was conducted, during which 38 were excluded for not being freely available, 18 were excluded for addressing a different population and not specifically master’s or doctoral students or alumni of *stricto sensu* graduate programs. Additionally, 24 studies that did not specify the context of graduate education in the health field were excluded, as well as 28 studies that did not encompass the concept, i.e., studies with a different methodological design and/or that did not address potentialities and/or challenges of *stricto sensu* graduate programs. The flowchart of the article selection process is presented below, as shown in Figure 1.

According to the search strategy developed, the languages, and the period used for article selection, from the 131 eligible studies, the final sample consisted of 23 studies. Regarding language, 12 (52.17%) studies were published in English,^19^^-^^20^^,^^28^^,^^31^^,^^33^^-^^34^^,^^36^^-^^41^ and 11 (47.82%) in Portuguese.^21^^-^^27^^,^^29^^-^^30^^,^^32^^,^^35^ Regarding the publication period, the studies were published between 2006 and 2022, originating from 11 different countries: ten (43.48%) from Brazil,^21^^-^^27^^,^^29^^,^^32^^,^^35^ three (13.04%) from the United States,^28^^,^^34^^,^^37^ two (8.69%) from Canada,^20^^,^^36^ two (8.69%) from Norway,^38^^,^^33^ one (4.35%) from Australia,^19^ one (4.35%) from France,^40^ one (4.35%) from New Zealand,^31^ one (4.35%) from the United Kingdom,^39^ one (4.35%) from Sweden,^41^ and one (4.35%) from Turkey.^30^

Only five studies used theoretical frameworks in their investigations. Of these, one^21^ (4.35%) study used the institutional analysis framework, one^29^(4.35%) used the sociopoetic framework, one^31^ used Vroom’s expectancy theory, one^35^ (4.35%) used the psychodynamics of work, and one^41^ (4.35%) used dialectical hermeneutics based on the fourth-generation evaluation model. Thus, 18^19^^-^^20^^,^^22^^-^^28^^,^^30^^,^^32^^-^^34^^,^^36^^-^^40^(78.26%) studies adopted a generic approach, meaning they did not specify the theoretical framework used; of these, eight^20^^,^^24^^,^^26^^,^^32^^,^^37^^-^^40^ (34.78%) used a mixed-methods approach. Regarding data collection, ten (41.66%) studies conducted semi-structured interviews,^19^^,^^21^^,^^23^^,^^27^^-^^28^^,^^30^^,^^34^^-^^35^^,^^40^^-^^41^ eight (34.78%) studies used open-ended/essay questions,^22^^,^^24^^,^^26^^,^^31^^-^^32^^,^^37^^-^^39^ three (13.04%) adopted focus groups,^20^^,^^25^^,^^33^ one (4.35%) study used a combination of focus groups and individual interviews,^36^ and one (4.35%) adopted the researcher group.^29^ The 23 studies included a total of 621 participants; 21 (91.30%) studies^19^^-^^25^^,^^27^^-^^36^^,^^38^^-^^41^ presented potentialities and challenges, one^26^ (4.35%) study presented only strengths, and one^37^ (4.35%) study presented only challenges of *stricto sensu* graduate programs in the health field. The characteristics of the studies are presented in Table 1.

**Table 1:** Characteristics of original studies included in the qualitative meta-synthesis

First Author, country, year	Journal	Design and data collection	Participants
Ellis LB. Australia 2006^19^	*Nurse Education Today*	Qualitative Semi-structured interview	Enrolled students in one of the professional doctorate programs (n=14). Nurses and midwives Nurses and midwives
Kearney R. Canada 2007^20^	*Academic Medicine*	Mixed Focus group	The focus group at the University of Calgary had five participants, and at the University of Alberta, seven participants (Canadian physicians with doctoral and master's degrees)
Depes VBS. Brazil 2013^21^	*Revista Gaúcha de Enfermagem*	Qualitative Exploratory Institutional Analysis Framework Semi-Structured Interview	Nine graduates, master's in nursing
Costa CMM. Brazil 2014^22^	*Saúde e Sociedade*	Qualitative Open-ended questions	20 students: 13 master's and 7 doctoral students, 18 from the Nutrition and Public Health Programs at the Public Health School, 1 from the USP Nursing School, and 1 from the Fernando Pessoa University, Portugal
Mendes VR. Brazil 2014^23^	*Revista Brasileira de Ciências do Esporte*	Qualitative Case study Semi-structured interview	Master's Programs in Physical Education, seven students enrolled in all research lines of the program and scholarship recipients
Souza LKCS. Brazil 2014^24^	*Revista de Nutrição*	Mixed Questionnaire and open-ended question	177 master's graduates from graduate programs in the field of Nutrition
Tavares CMM. Brazil 2014^25^	*Revista da Rede de Enfermagem do Nordeste*	Qualitative Exploratory Case study Focus group	12 newly admitted students in the Professional Master's Program in Nursing
Geremia HC. Brazil 2015^26^	*Psicologia: Ciência e Profissão*	Mixed Questionnaire and essay questions	29 psychologists, students in the master’s program in psychology at a Brazilian federal university
Galdino MJQ. Brazil 2016^27^	*Revista de Enfermagem UFPE online*	Qualitative Descriptive Exploratory Semi-structured interview	25 master's students in nursing from a Brazilian public university
Remich R. United States 2016^28^	*Academic Medicine: Journal of the Association of American Medical Colleges*	Qualitative Semi-structured interview	22 students in a biomedical doctorate program
Ferreira RE. Brazil 2018^29^	*Revista de Enfermagem UFPE online*	Qualitative Descriptive *Sociopoética* A research group with aesthetic experimentation working with the five senses	12 nurses enrolled in the academic and professional master's programs
Ünal A. Turkey 2018^30^	*Acta Paulista de Enfermagem*	Qualitative Descriptive Semi-structured interview	16 individual interviews with doctoral students enrolled at the Institute of Health Sciences, Nursing Schol
Alamri Y. New Zealand 2019^31^	*BMC Medical Education*	Qualitative Exploratory Vroom’s Expectancy Theory Open-ended questions	22 former and current MBChB/PhD students, Dean's Office, Otago Medical School, Dunedin, New Zealand
Engstrom EM. Brazil 2020^32^	*Ciência e Saúde Coletiva*	Mixed Questionnaire and essay questions	22 graduates of the Professional Master's in Primary Health Care in the Municipality of Rio de Janeiro, Brazil.
Kjellaas S. Norway 2020^33^	*Nordic Journal of Nursing Research*	Qualitative Descriptive Focus group	18 participants: fifteen had a master's in clinical nursing, and three had a master's in community health, public health, and nutrition/health
May JT. United States 2020^34^	*Journal of Professional Nursing*	Qualitative Descriptive Semi-structured interview	4 nursing students with a dual DNP doctorate
Moreira DA. Brazil 2020^35^	*Revista Brasileira de Enfermagem*	Qualitative Integrated single case study Work psychodynamics Semi-structured interview	23 students participated: 12 master's and 11 doctoral students in a *stricto sensu* Nursing Graduate Program in Brazil
Allard E. Canada 2021^36^	*Nurse Education in Practice*	Qualitative Focus group / Individual Semi-structured interview	15 doctoral students in nursing at a Canadian school.
Chakraverty D. United States 2022^37^	*BMC Medical Education*	Mixed Questionnaire and essay questions	9 MD-PhD students and residents (five men, four women; four white, five from other ethnic backgrounds) also completed an interview.
Darj E. Norway 2022^38^	*International Journal of Environmental Research and Public Health*	Mixed Questionnaire and essay questions	39 doctoral graduates from the Norwegian School of Global Health Research
Hampshaw S. United Kingdom 2022^39^	*Nurse Education in Practice*	Mixed Questionnaire and essay questions	47 nurses with a doctorate (including 33 adult health nurses, 4 mental health nurses, 6 pediatric nurses, 2 health visitors, and 2 midwives)
Met N. France 2022^40^	*Journal of Nursing Management*	Mixed Questionnaire and Semi-structured interview	45 semi-structured interviews with nurses, 10 interviews with health managers and head nurses
Nylander E. Sweden 2022^41^	*New Review of Academic Librarianship*	Qualitative Exploratory Dialectical hermeneutics based on the fourth generation evaluation model Semi-structured interview	Twelve open interviews with doctoral students and their advisors at the Research School of Health and Welfare in Jönköping, Sweden

The overall quality of the studies included in the meta-synthesis was satisfactory (Table 2). All^19^^-^^41^ demonstrated coherence between objectives, methodology, research design, recruitment strategy, research question approach, clear results, and contributions. However, it is worth noting that two^22^^-^^23^ (8.69%) did not describe the ethical aspects of the research, and in most cases, 13^19^^,^^21^^,^^22^^-^^27^^,^^31^^,^^36^^,^^38^^-^^40^ (56.52%), reflexivity aspects were not mentioned, i.e., the researcher's critical analysis of their relationship with participants and the possibility of bias. Regarding the relationship between the researcher and participants, nine^20^^,^^28^^-^^30^^,^^32^^-^^33^^,^^35^^,^^37^^,^^41^(39.13%) were classified as partially considered, as the study mentioned that the researcher conducted data collection; however, aspects of reflexivity were not cited. It is worth noting that only one^34^ (4.35%) study adequately addressed the relationship between the researcher and participants. Finally, two^24^^,^^26^ (8.69%) studies did not describe the data analysis process.

**Table 2:** Results of the quality assessment of articles according to CASP

Questions	Yes	Partially	No
Were the research objectives clearly reported?	^19^^-^^41^		
Was the qualitative methodology appropriate?	^19^^-^^41^		
Was the research design suitable for achieving the proposed objectives?	^19^^-^^41^		
Was the recruitment strategy appropriate for the research objectives?	^19^^-^^41^		
Were the data collected in a way that addressed the research question?	^19^^-^^41^		
Was the relationship between the researcher and participants properly considered?	^34^	^20^^,^ ^28^^–^^30^^,^ ^32^^,^ ^33^^,^ ^35^^,^ ^37^^,^ ^41^	^19^^,^ ^21^^,^ ^22^^–^^27^^,^ ^31^^,^ ^36^^,^ ^38^^–^^40^
Were ethical issues considered?	^19^^–^^21^^,^ ^24^^–^^26^^,^ ^28^^–^^41^	-	^22^^–^^23^
Was the data analysis sufficiently rigorous?	^19^^–^ ^20^^–^ ^21^^–^ ^22^^–^ ^23^^,^ ^25^^,^ ^27^^–^ ^41^	-	^24^^–^^26^
Were the results clearly reported?	^19^^–^^41^		
Did the research provide contributions?	^19^^–^^41^		

### Knowledge Synthesis

**Figure 2:**
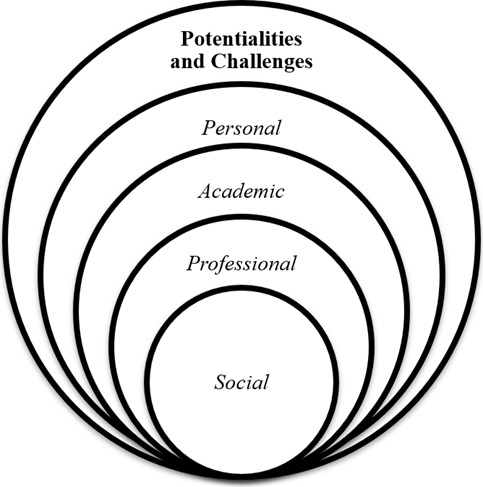
Presentation of the knowledge synthesis based on the potentialities and challenges identified

The knowledge synthesis allowed for categorizing potentialities and challenges and subcategorizing them into four domains: personal, academic, professional, and social. All these domains correspond to both potentialities and challenges, as they enable their articulation according to the studies analyzed in this meta-synthesis. The categorization and subcategorization are presented in Figure 2.

From the schematic representation of the results, as shown in Figure 2, it is understood that potentialities and challenges, although subdivided into personal, academic, professional, and social domains in this meta-synthesis, are not necessarily isolated, as they may integrate depending on the situation. For example, a high level of skills, knowledge, and experience, while categorized as a personal strength, also supports academic strength/professional. Thus, each categorization of potentialities and challenges can intersect in specific situations and reverberate across other domains. For instance, academic strength derived from research development can impact social strength. The same applies to challenges. Below, Tables 3 and 4 will describe, based on the selected studies, the strengths and challenges arising from *stricto sensu* graduate programs in the health field. According to the analysis by the authors of this meta-synthesis, the potentialities and challenges identified in the studies are subdivided into personal, academic, professional, and social categories:

**Table 3:** Personal, academic, professional, and social potentialities

Personal potentialities
Acquired knowledge^21^^,^^30^^,^^35^^,^^40^
Confidence^21^^,^^39^^,^^41^
Reaffirmation of ethical values^21^^,^^25^
Critical thinking^22^^,^^25^^,^^39^
Achievement of a dream^27^^,^^29^
Autonomy^29^^,^^31^
Pride and satisfaction in completing the program^27^^,^^39^
Ability to develop arguments^19^
Intellectual reward^20^
Capacity for personal transformation; self-esteem, belief in one’s potential; overcoming; recognition of limitations; solidarity^21^
Tolerance and resilience^23^
Gender as a source of pride and strength^28^
Empowerment^29^
Self-recognition^27^
High level of acquired skills^30^
Motivation; maturity^31^
Support from colleagues, family, and friends^36^
Maturity; satisfaction in achieving individual goals; self-taught ability and informational competence^41^
Academic potentialities
Advanced development of research skills^19^^,^^31^
Participation in research projects^19^^,^^40^
Qualification for teaching^24^^,^^35^
Advancement of research in the field of study^19^
Motivation for a research career^20^
Mobilization of the educational process^21^
Quality of education, derived from duration of stay, student dedication, and graduate commitment^23^
High qualification of faculty; scientific improvement^24^
Importance of the advisor^26^
Knowledge production; interdisciplinary nature of the program^29^
Ability to conduct research and publish findings^30^
Experience^31^
Participation in courses; recognition of work developed; university reputation; research development^35^
Diverse pedagogical strategies: problem-based learning, active teaching methodologies; availability and harmonious relationship with coordination and faculty^32^
Educational, interesting, exciting, and enriching potential of scientific investigations^33^
Advisor’s conduct characterized by accessibility, feedback, and investment in students^34^
Academic development^40^
Academic networking^38^
Fulfillment of educational roles^39^
Professional potentialities
Connection between research and professional practice, contributing to service delivery^19^^,^^21^^-^^22^^,^^25^^,^^29^^,^^31^^,^^39^
Salary increase^22^^,^^26^^,^^33^
Career planning^26^^,^^31^
Encouragement to pursue a doctorate at work^22^^,^^40^
Professional development^27^^,^^30^
Problem-solving ability^30^^,^^33^
Professional credibility; clinical leadership^19^
Professional recognition^22^
Institutional recognition^25^
Pursuit of a teaching and research career; search for qualification^26^
Job opportunities^32^
Enhanced competence; greater professional confidence; effective services; decision-making and responsibility in disseminating knowledge to colleagues and supervisors; assistance in development projects and tasks at work; evidence-based practice^33^
Career progression; working in Higher Education Institutions^39^
Teaching in healthcare services^40^
Social potentialities
Social recognition and appreciation for undertaking a *stricto sensu* graduate program^26^^,^^39^
Encouraging individuals within one’s social circle to pursue the program^21^
Social representation^28^
Social contribution derived from the studies conducted^30^

Regarding challenges, which are categorized as personal, academic, professional, and social, the results are presented in Table 4:

**Table 4:** Personal, academic, professional, and social challenges

Personal challenges
Difficulty balancing the program with family demands^20^^,^^36^^,^^41^
Personal demands^20^^,^^27^
Stress^39^^,^^41^
Family support; time management^20^
Pursuing only the degree without ensuring actual growth opportunities^22^
Questions about the relevance of the program; problematic phase transitions and psychological suffering, including dissatisfaction, physical and emotional exhaustion^31^
Insecurity, feelings of disqualification; lack of family recognition; minority status (race, social condition)^37^
Being new to the country where the graduate program is conducted^38^
Disappointment, lack of support^39^
Confusion, frustration^41^
Academic challenges
Lack of funding^19^^-^^20^^,^^31^
Demands for productivity; competition among students^23^^,^^35^
Tight deadlines^24^^,^^35^
Conducting solitary work^29^^,^^35^
Lack of experience and skills of the advisor^36^^,^^37^
Traditionalism, resistance to change; difficulties in data collection; challenges obtaining ethical approval; scarcity of advisors^19^
Need for institutional support; need to balance teaching demands^20^
Overload^23^
Difficulty in publishing; pressures; overemphasis on productivity hindering more qualified training; insufficient preparation for teaching^24^
Gender bias and challenges persisting in an academic career^28^
Gaps in education and curriculum; deficiencies in the program; advisor’s direction differing from the student’s proposed research; need for innovation^30^
Administrative obstacles; access to human support, colleagues, informed supervisors, and specialized resources^31^
Evaluation strategies used; superficial presentation of research methodology^32^
Challenges related to the research process^33^
Absent, apathetic, uninterested advisors^34^
Demands; coursework; lack of recognition by the advisor^35^
Exams and graduate program activities^36^
Hostile treatment by peers; fear of evaluation; transitioning between programs (pre-clinical and research training); lack of belonging to the research community^37^
Need for continuous support; COVID-19 pandemic^38^
High performance demands; constant need to seek permission from the advisor; lack of awareness regarding informational strategies offered by the graduate program^41^
Professional challenges
Balancing graduate studies with work^23^^,^^27^^,^^36^^,^^39^^,^^41^
Difficulty integrating clinical and research careers^20^^,^^37^
Administrative routines; need for organizational support at work^20^
Concern that learning may not lead to changes in care practices^21^
Low return on investment; limited opportunities for an academic career in the professional sphere^22^
Lack of time to complete the program^23^
Hopelessness regarding the contribution of graduate studies to workplace service and lack of support at work; discouragement toward the program within the work context and with supervisors^25^
Uncertainties arising from career planning changes; common language barriers with clinicians^30^
Devaluation of the degree within the organization; need to improve university-hospital cooperation utilizing this qualification by organizations; graduate studies perceived as a threat by supervisors and colleagues^33^
Difficulties in forming a professional identity^37^
Difficulty transitioning from student to employee^38^
Career advancement difficulties, status and/or salary; lack of opportunities to utilize skills developed during graduate studies^39^
Lack of opportunities, coupled with low visibility and recognition of programs within healthcare organizations; absence of assigned tasks; lack of suitable positions, poor professional integration^40^
Social challenges
Prestige loss^19^
Lack of societal recognition and appreciation^33^^,^^39^

## Discussion

This review enabled the synthesis of potentialities and challenges from the perspective of graduate students/alumni of *stricto sensu* graduate programs in the health field. This approach allowed for a comprehensive description considering participants' experiences within the graduate education context. Two main categories were developed: potentialities and challenges, subdivided into common subcategories encompassing personal, academic, professional, and social potentialities/challenges. Based on the findings of this meta-synthesis, it can be inferred that *stricto sensu* graduate programs in health present a rich landscape of possibilities while also facing obstacles that impact this journey and need to be overcome.

Regarding academic potentialities, Higher Education Institutions (HEIs) substantially contribute to knowledge advancement and addressing socioeconomic challenges faced by society.^42^ Within the graduate education context, master's and doctoral training provides intellectual development that can serve educational institutions while also contributing a skilled workforce for societal and economic development.^43^ In academia, scientific productivity is essential for ensuring stability and promoting progress in the field. In this sense, graduate programs play a crucial role by significantly contributing to academic performance and advancing scientific productivity,^44^ benefiting society and promoting academic and personal skill development.^45^ To effectively train human resources for the healthcare system, it is essential to equip students with appropriate teaching and evaluation methods, as well as develop necessary competencies for educational planning and assessment.^46^This training process presents professional potentialities including career enhancement and improved professional practice,^3^ as also highlighted in the findings of this meta-synthesis. A study from the USA with plastic surgeons revealed that these professionals pursue advanced degrees to enable scientific development. For these professionals, a master’s degree is associated with greater academic activity, research funding, career development, publications, citations, and leadership positions.^47^

Thus, motivations for pursuing graduate studies include financial stability, prestige, work-life balance, and the development of critical thinking skills.^48^ Such skills reinforce the personal, academic, professional, and social potentialities that are developed during the *stricto sensu* graduate training process. In this regard, it is worth noting that graduate students’ perceptions of the training process are essential elements for evaluating the quality of education at this academic level,^49^ particularly through a qualitative approach. In this direction, graduate programs that provide effective guidance and financial support can produce well-prepared researchers, capable of taking on leadership roles and postdoctoral positions. In the health field, specifically in nursing, research enables solutions for clinical contexts, including new strategies and models of patient care. Thus, nurses with doctorates can also prepare the next generation of professionals through academic leadership and as members of faculty and mentors in course curricula,^50^ expanding career opportunities as verified in the professional potentialities.

According to a Dutch study conducted with 391 PhD candidates, although most participants experienced fair, open, integral, reliable, and freedom-promoting evaluation processes in their research environments, many reported facing challenges such as lack of time and support, insufficient supervision, and witnessing questionable research practices.^51^ Regarding academic challenges, questionable authorship practices prevail among early-career researchers and seem to be reinforced by coercive power dynamics and dominant norms in some research cultures, particularly in natural, technical, and medical sciences.^52^ A Brazilian study conducted with 38 master’s students and 35 doctoral candidates in the health field showed that the relationship with the supervisor can be dual, depending on the profile and conduct of this professional. Thus, when the supervisor effectively fulfills their role, they guide the student in conducting research and navigating the graduate program, showing empathy, addressing intellectual and socio-emotional dimensions, and serving as a career role model. However, supervisors with obstructive behaviors, such as hierarchical relationships, communication difficulties, neglect in supervision, and resistance to building rapport, impact the student’s academic-professional development and the execution of research..^53^

Similar to the results of this study, U.S.-based research involving 568 doctoral students from 53 nursing schools revealed that barriers faced by students are centered on faculty-related issues, time management, insufficient preparation for dissertation research, financial barriers, and the impact of COVID-19.^54^ During the COVID-19 pandemic, graduate students faced an increased risk of mental health problems,^55^ becoming a challenge as presented in the results of this meta-synthesis. Students were particularly affected by changes in daily life due to lockdowns, travel restrictions, remote teaching, financial difficulties, and reduced social interactions.^56^ Additionally, excessive workload, along with physical and mental exhaustion, can develop or exacerbate disorders among graduate students in the health field. Thus, the poor mental health of graduate students has become increasingly concerning, representing a personal challenge that reverberates in academic, professional, and social spheres.^57^ In this direction, a Chinese study unveiled that academic stressors relate to high supervisor expectations, the need for self-discipline, peer comparison in academia, difficulties in changing research directions/academic disciplines, uncertainties about future careers, language barriers, challenges in living in another city/country, and limited social interaction with others.^58^

Thus, various personal and family demands and responsibilities are evident-personal challenges may also be related to professional ones, such as the need to balance work with coursework, often without organizational encouragement or support. Many organizations underestimate doctorates in clinical settings, resulting in the loss of PhD holders from clinical practice to academic environments.^39^ Therefore, there is a need for continuous efforts toward research cultures and infrastructures with appropriate career paths and positions for PhD professionals in healthcare organizations.^59^ As Cassiano and colleagues^57^ explain, regarding social challenges, the lack of societal recognition of the degree and research development impacts the motivation of health field graduate students, especially since society often does not understand what graduate studies entail. According to Caldas’ reflections,^60^ the activities performed by graduate students in higher education deserve to be valued and recognized as genuine work, especially given their intense productivity and dedication. The experience during graduate studies is characterized by ambivalence, as also shown in our study, where students report facing severe challenges at physical, emotional, and relational levels, but at the same time, recognize that this environment can be a source of growth, maturity, and happiness. This duality of feelings shows that graduate students experience both sadness and uncertainty, as well as satisfaction and joy during their academic journey.^60^This suggests that the graduate journey is full of personal, academic, professional, and social challenges, but also moments of overcoming, achievements, and rewards that enrich the experience and represent potentialities in the personal, academic, professional, and social domains of students.

By recognizing this complexity of challenges faced by graduate students, it is possible to improve the support and monitoring provided by educational institutions, aiming to create a healthier and more welcoming environment. Furthermore, it is important to value the achievements and efforts of these students, recognizing them as an essential part of scientific knowledge construction and society as a whole. A limitation of this study is the language criterion, as only studies in Portuguese, English, and Spanish were included. Few studies using qualitative theoretical-methodological frameworks were identified. However, as strengths, the extensive 20-year period used for article recruitment should be considered. Moreover, the findings encompassed 11 different countries worldwide, enabling the extension of these discoveries and synthesizing knowledge from a global perspective.

Conclusion. The analysis from this meta-synthesis allows us to conclude that *stricto sensu* graduate programs in the health field have potentialities such as knowledge acquisition, scientific advancement, career opportunities, and social contribution, but also face challenges like emotional vulnerabilities, difficulties in balancing and managing time, and lack of integration with services. The knowledge produced proves useful for understanding the strengths and challenges of *stricto sensu* graduate programs in the health field, offering a worldwide, comprehensive, and in-depth view of the current research landscape in this area. It is therefore important to strengthen these potentialities and mitigate the challenges, especially through institutional support. It is important that training centers create and manage strategies to help graduate students address, especially, the challenges present in this process. Moreover, it is pertinent to conduct further investigations on the topic, highlighting qualitative studies that allow for an understanding of subjectivities in this context.

We suggest, for future studies, the development of additional qualitative research on the topic, expanding to other participants in this academic field and other graduate areas beyond health, as well as utilizing appropriate qualitative theoretical-methodological frameworks for each study. By understanding the perspectives and perceptions of those involved, it is possible to enhance academic training strategies, encourage scientific excellence, and provide a more favorable environment for the personal, academic, professional, and social development of *stricto sensu* graduate students.
